# Characterizing and mitigating protocol-dependent gene expression bias in 3′ and 5′ single-cell RNA sequencing

**DOI:** 10.3389/fbinf.2026.1931243

**Published:** 2026-10-08

**Authors:** Valeryia Shydlouskaya, S. M. Mansour Haeryfar, Tallulah S. Andrews

**Affiliations:** 1 Department of Microbiology and Immunology, Schulich School of Medicine and Dentistry, Western University, London, ON, Canada; 2 Collaborative Specialization in Machine Learning in Health and Biomedical Sciences, Western University, London, ON, Canada; 3 Department of Oncology, Schulich School of Medicine and Dentistry, Western University, London, ON, Canada; 4 Department of Medicine, Division of Clinical Immunology & Allergy, Schulich School of Medicine and Dentistry, Western University, London, ON, Canada; 5 Department of Surgery, Division of General Surgery, Schulich School of Medicine and Dentistry, Western University, London, ON, Canada; 6 Department of Biochemistry, Schulich School of Medicine and Dentistry, Western University, London, ON, Canada; 7 Department of Computer Science, Western University, London, ON, Canada

**Keywords:** 3′ and 5′ protocol bias, batch effect correction, benchmarking, data integration, differential gene expression, gene expression normalization, single-cell RNA sequencing

## Abstract

Single-cell RNA sequencing (scRNA-seq) has enabled large-scale characterization of cellular heterogeneity; yet, integrating datasets generated through different library preparation protocols remains challenging. For instance, comparisons between 10X Genomics 3′ and 5′ chemistries are complicated by protocol-dependent technical biases imposed by differences in transcript end capture and amplification. While normalization, and often batch correction, is an integral step in preprocessing scRNA-seq datasets, it remains unclear which correction is most appropriate, or even necessary, for reliable cross-protocol comparisons. Here, we systematically characterize protocol-related expression differences using matched 3′ and 5′ scRNA-seq data from 36 subjects across six datasets spanning multiple tissues. We find that gene expression discrepancies are not pervasive across the whole transcriptome but are instead driven by a relatively small, reproducible subset of protocol-biased genes. These protocol-specific differences were not primarily explained by GC content or gene length. Excluding the affected genes improves cross-protocol concordance, indicating that most genes are directly comparable without aggressive correction. We then benchmark eleven commonly employed normalization approaches and show that while several methods, such as fastMNN, improve statistical alignment when cell populations are well matched, they can distort gene-level signals and inflate differential expression in biologically realistic settings with incomplete cell-type overlap. Taken together, our results demonstrate that protocol bias between 3′ and 5′ scRNA-seq is limited in scope and that targeted handling of biased genes presents an alternative approach to normalization or batch correction strategies when integrating and comparing data across protocols.

## 1 Introduction

Single-cell RNA sequencing (scRNA-seq) is a recent technology that has become an invaluable tool for gene expression analysis with single-cell resolution. In recent years, this technique has been widely used to address key biological questions of cell heterogeneity in different tissues and disease states, including but not limited to cell differentiation, immune responses, and tumor heterogeneity (Zheng et al., 2018; Cao et al., 2019; 2020; Wagner et al., 2019; Yost et al., 2019; Gu et al., 2024). Current scRNA-seq technologies require single cell isolation, capturing or tagging of mRNA with unique barcodes, and cDNA synthesis, fragmentation and amplification to generate sequencing libraries consisting of ∼300-base-pair-long barcoded transcript fragments. Due to the necessity of adding unique molecular identifiers (UMIs) during the reverse transcription step to reduce amplification noise (Kivioja et al., 2012; Ziegenhain et al., 2017), barcodes are attached to only one end of each transcript, either 3′ or 5′, which means only the fragments containing the appropriate end are amplified and sequenced.

Originally, scRNA-seq protocols included barcodes on the poly(dT) oligonucleotides used to capture the mRNA molecules. As a result, UMIs were attached to the 3′ end of each transcript during reverse transcription (Macosko et al., 2015; Zilionis et al., 2017). However, this approach was far from ideal in interrogating immune landscapes shaped by T and B lymphocytes since only the constant region of recombined T-cell receptors (TCRs) and B-cell receptors (BCRs) could be sequenced. To enable single-cell resolution characterization of the recombined and variable portions of TCRs and BCRs, protocols were altered to instead include UMIs and cell-specific barcodes in switch oligonucleotides, thereby adding them to the transcripts’ 5′ end (Pai and Satpathy, 2021). Accordingly, many newer scRNA-seq studies, particularly those focusing on immune landscapes and responses, use this 5′ scRNA-seq protocol (van der Wijst et al., 2021; Gao et al., 2022; Oliver et al., 2024). It is noteworthy, however, that this approach has complicated the efforts to generate comprehensive atlases or perform meta-analyses due to technical biases in gene expression measured with 3′ and 5′ technologies (Tran et al., 2020).

Previous benchmarking studies have examined the efficacy of different computational methods at integrating various protocols in a shared lower-dimensional space (Tran et al., 2020; Luecken et al., 2022; Zhong et al., 2025). However, these approaches are limited to improving the results of clustering, trajectory inference, or other algorithms that only utilize a lower-dimensional representation of scRNA-seq data. They do not enable comparisons of gene expression levels between different cell populations or biological conditions. To date, no systematic comparisons have been conducted to evaluate available approaches for gene-expression space normalization to correct technical differences across scRNA-seq protocols.

Studies assessing protocol-related differences between scRNA-seq and single-nucleus RNA sequencing (snRNA-seq) reveal potential challenges of cross-protocol correction. While it has been shown that certain technical effects can be partially corrected through simple adjustments like regressing out gene length (Lake et al., 2017; Bakken et al., 2018), these approaches do not fully reconcile scRNA-seq and snRNA-seq data due to confounding biological effects, such as cytoplasmic post-transcriptional regulation (Guo et al., 2010) and dissociation-associated stress responses that are more common in scRNA-seq than in snRNA-seq (Denisenko et al., 2020). In contrast, differences between 10X Genomics 3′ and 5′ scRNA-seq protocols are simpler and are thought to primarily result from technical variations in transcript end capture during reverse transcription and downstream amplification (Aird et al., 2011). As these protocol-related effects are not expected to reflect genuine biological differences between cells, they may be well suited to correction using existing normalization and batch correction approaches. Nonetheless, it remains unclear to what extent correction is necessary and, if so, which methodologies are most effective at reconciling 3′ and 5′ scRNA-seq data without distorting biologically meaningful signals (10X Genomics, 2024).

There are many widely used normalization and batch correction methods that explicitly operate at the level of gene expression values and do not require cell type label information (Tran et al., 2020; Luecken et al., 2022). These include linear adjustment methods, such as ComBat (Johnson et al., 2007) and limma (Ritchie et al., 2015); mutual nearest neighbor–based approaches, including R-based mnnCorrect (Haghverdi et al., 2018) and fastMNN (Haghverdi et al., 2018) as well as Python-based Scanorama (Hie et al., 2024), which adjust expression values by aligning shared cellular neighborhoods across batches; and model-based, gene-wise normalization frameworks such as M3Drop (Andrews and Hemberg, 2019b) and SCTransform (Hafemeister and Satija, 2019), which use negative binomial models to account for depth-dependent technical noise and produce Pearson residuals that can be used as corrected expression values; and deep generative models such as scVI (Lopez et al., 2018) and scArches (Lotfollahi et al., 2022), which learn latent representations of scRNA-seq data and which can reconstruct normalized expression estimates while accounting for technical variation. However, it is not known whether any of these approaches can accurately correct protocol differences at the level of gene expression.

In this manuscript, we characterized protocol-dependent gene expression differences between 3′ and 5′ scRNA-seq and evaluated the performance of existing normalization and batch correction methods. Using data from 36 subjects from six publicly available datasets with matched 3′ and 5′ scRNA-seq, we first identified a subset of genes whose expression is consistently biased between these chemistries and examined their recurrence, expression distribution, biological functions, cell-type specificity, and transcript features. We then evaluated eleven selected correction methods for their ability to mitigate the noted biases. Performance was assessed by comparing the similarity of cell-type-specific expression profiles between 3′ and 5′ data, evaluating the degree of mixing between 3′ and 5′ samples following correction, and examining the agreement in cell-type-specific marker genes. Finally, we tested the top-performing methods in a synthetic use-case designed to integrate partially overlapping 3′ and 5′ datasets. Based on these results, we show that excluding the genes strongly affected by protocol differences was the most effective approach among those tested for reducing false findings when comparing expression levels across 3′ and 5′ scRNA-seq datasets.

## 2 Methods

### 2.1 Dataset acquisition

Six human datasets were identified from the CELLxGENE database that contained the same biological samples from 36 subjects profiled using both 5′ and 3′ Chromium scRNA-seq (Supplementary Table S1) (Park et al., 2020; Jardine et al., 2021; Salcher et al., 2022; Suo et al., 2022; CZI Cell Science Program et al., 2025; Andrews et al., 2024; De Simone et al., 2025). All processed datasets and their matching metadata, including cell-type annotations, were downloaded. For each dataset, we identified subjects with matched 3′ and 5′ scRNA-seq samples generated from the same tissue using the same cell isolation strategy, when applicable. To ensure comparable sample composition and quality between 5′ and 3′ data, subjects with the most extreme discrepancy in their frequency of any cell type between 3′ and 5′ samples, where the cell type in question represented at least 5% of the total dataset, were excluded, with the aim of limiting these discrepancies to <25% where possible (Supplementary Figure S1). We used the cell-type annotations provided by the dataset authors. Datasets with fine-grain annotations that identified more than 20 cell types, many of which were highly similar, were manually re-annotated at a coarser level, accounting for cell-type frequencies (Jardine et al., 2021; Salcher et al., 2022; Suo et al., 2022; Andrews et al., 2024) (Supplementary Tables S2-S5). Cells from rare cell types (<50 cells in either 3′ or 5′ samples) were removed to prevent sparsely represented populations from biasing normalization and integration performance assessments.

### 2.2 Data preprocessing and highly variable genes

Datasets were split into pairs of matching 3′ and 5′ data for each subject and processed separately, preventing genetic differences between subjects from confounding the results. Raw counts were library-size normalized to 10,000 counts per cell and natural-log transformed using Seurat (v5.1.0) (Hao et al., 2024) with a pseudocount of 1. The top 3,000 highly variable genes (HVGs) were identified using a vst model (Hafemeister and Satija, 2019). All samples originating from the same tissue within each dataset were combined, and a consensus set of 3,000 HVGs was identified using Seurat.

### 2.3 Identification of protocol-biased genes

To identify genes that were consistently differentially expressed between the 10X 3′ and 10X 5′ assays, we conducted differential gene expression analysis for each subject across all cells using a Wilcoxon rank-sum test implemented in Seurat (v5.1.0). We then calculated the cumulative number of overlapping differentially expressed genes (DEGs) across subjects at three different *p*-value thresholds (*p* < 1e^−5^, 1e^−50^, and 1e^−100^). Consensus biased genes were identified at the “elbow” in the cumulative distribution. Uniform Manifold Approximation and Projection (UMAPs) were generated using default parameters with 30 principal components before and after the removal of consensus biased genes. Integration efficacy was assessed using the Seurat MixingMetric (Stuart et al., 2019) with a neighborhood size of 150 cells to balance local and global scales. Consequently, a consensus list of 867 protocol-biased genes (significant in at least five subjects at 1e^−50^) was selected for downstream analyses. To assess the contribution of these genes to platform differences, we computed cosine similarity scores between 3′ and 5′ data using the biased gene list and three replicates of randomly sampled gene sets of equal size and expression level composition.

### 2.4 Validation of protocol-biased genes in additional matched datasets

To evaluate whether the 867 protocol-biased genes generalized beyond the samples used for discovery and benchmarking, seven additional matched 3′ / 5′ subject samples were retained for validation of the protocol-biased gene set (Supplementary Table S1). Three validation subjects were sourced from one novel kidney dataset (Acera-Mateos et al., 2026). Additionally, four subject samples from kidney, skin, and liver were retained from the Suo et al. dataset (Suo et al., 2022) considered during the primary analysis but excluded from biased-gene discovery because of quality-control considerations. These validation subjects were not used to identify the 867 protocol-biased genes, select the recurrence threshold, or tune any biased-gene filtering criteria. Each subject was processed using the same workflow as the primary benchmark datasets with the exception of balancing the cell numbers between the 3′ and 5′ . We then assessed whether removal of the predefined 867 genes improved concordance between matched 3′ and 5′ data using UMAP visualization, the Seurat MixingMetric, and cosine similarity between matched 3′ and 5′ cell-type expression profiles. We also confirmed that the number of differentially expressed genes among the 867-gene biased set, identified using a Wilcoxon rank-sum test at FDR <0.05, was comparable to that observed in the discovery datasets.

### 2.5 Defining detected genes

Genes were considered detected if they had non-zero counts in >1% of cells in at least one of the six benchmark datasets. The reference gene universe and gene biotypes were obtained from the Cell Ranger GRCh38-2024-A GTF annotation. Undetected genes were classified by gene biotype. Gene Ontology Biological Process enrichment of undetected protein-coding genes was performed using clusterProfiler (v4.14.6), with all annotated protein-coding genes as the background. Terms with FDR <0.05 and fold enrichment >2 were retained and manually grouped into broad biological categories based on GO term descriptions.

### 2.6 Characterization of protocol-biased genes

To characterize the expression distribution of the 867 consensus protocol-biased genes, normalized expression values were aggregated by subject with Seurat (v5.1.0). For each subject, genes with zero expression were assigned to a separate Expression 0 category. Genes with non-zero expression were ranked and divided into four expression-rank quartiles: Q1, lowest 25%; Q2, 25%–50%; Q3, 50%–75%; and Q4, highest 25%. Additionally, the proportion of immune cells (i.e., leukocytes) was calculated for each subject.

For cell-type-specificity analysis, cell types represented by fewer than 5 cells were excluded. A gene was classified as non-immune specific if it was detected in ≥30% of cells in at least one non-immune cell type and in <10% of cells in every evaluated immune cell type.

GO (Biological Process, Cellular Component, and Molecular Function) over-representation analysis of the 867 protocol-biased genes was performed using clusterProfiler (v4.14.6), with all detected genes as the background.

When protocol-biased genes were removed, it was done prior to any preprocessing to prevent any potential influence on preprocessing steps.

### 2.7 Transcript-end GC content and gene length

For each gene, protein-coding transcript isoforms were obtained from EnsDb.Hsapiens.v86 (v2.99.0), and the isoform with the greatest summed exonic length was selected as the representative transcript. Its exonic sequences were then obtained from BSgenome.Hsapiens.UCSC.hg38 (v1.4.5) and concatenated in transcript orientation, excluding introns and the post-transcriptionally added poly(A) tail. GC fraction was calculated separately for the first and last 250, 500, and 1,000 nucleotides of the spliced transcript, or across the complete transcript when it was shorter than the specified window. Furthermore, representative transcript length was defined as the summed exonic length of the longest annotated protein-coding transcript for each gene.

### 2.8 Intronic vs. exonic quantification

To test whether alignment with or without intronic reads affected the detection of protocol-biased genes, we selected one subject-level comparison with approximately 5,000 cells in each of the 3′ and 5′ samples. The CD45^+^ spleen sample from individual F45 in the Suo dataset (Suo et al., 2022) was selected for this analysis. Raw FASTQ files were obtained from ArrayExpress (E-MTAB-11343) and remapped using Cell Ranger (v7.0.1) with the “--include-introns” flag set to true or false. Resulting expression matrices were filtered to remove cells with fewer than 500 detected genes or >10% mitochondrial expression in either quantification. These were further filtered using the same criteria as above to ensure that identical cells and cell-type annotations were included in both the exonic-only and exonic-plus-intronic matrices. Differential expression between matched 3′ and 5′ libraries was then repeated using the same Wilcoxon rank-sum workflow described above. We compared the total number of significant 3′ / 5′ DE genes, the number of significant genes from the predefined 867-gene list, and the directionality of biased genes under exonic-only and exonic-plus-intronic quantification.

### 2.9 Data normalization

Each subject pair of 3′ and 5′ data was normalized using the following approaches, which are compatible with correcting the gene expression values and do not require cell type information (Supplementary Table S6):

***SCTransform-regression***: Normalization was performed using sctransform (v0.4.1) (Hafemeister and Satija, 2019) implementation with default parameters on merged 3′ and 5′ data, with assay type (3′ vs. 5′) included as a covariate in the regression model.

***SCTransform-single***: sctransform (v0.4.1) (Hafemeister and Satija, 2019) with default parameters was applied independently to 3′ and 5′ datasets, which were subsequently merged.

***M3Drop***: A depth-adjusted negative binomial (DANB) model was fit separately to 3′ and 5′ data using M3Drop (v1.32.0) (Andrews and Hemberg, 2019b). Pearson residuals derived from fitted models were used as corrected expression values.

***mnnCorrect***: Log-normalized expression values from merged 3′ and 5′ datasets were corrected at the expression level using mnnCorrect from batchelor (v1.22.0) (Haghverdi et al., 2018). Due to computational constraints, this method was applied only to HVGs and consensus protocol-biased genes.

***fastMNN***: Normalization was performed as above using the *fastMNN* algorithm from batchelor (v1.22.0) (Haghverdi et al., 2018).

***Scanorama***: Log-normalized expression values from matched 3′ and 5′ datasets were corrected using the *correct_scanpy* function from Scanorama (v1.7.4) (Hie et al., 2024). Correction was performed with 3′ and 5′ samples provided as separate batches.

***ComBat***: Log-normalized expression values from merged 3′ and 5′ datasets were corrected using the parametric implementation of *ComBat* in sva (v3.54.0) (Johnson et al., 2007), which adjusts both the mean and scale of batch effects.

***Limma***: Log-normalized expression values from merged 3′ and 5′ datasets were corrected using the *removeBatchEffect* function from limma (v3.62.2) (Ritchie et al., 2015), which fits a linear model to each gene and removes systematic differences attributable to batch effects while preserving overall expression structure.

***Z-transformation***: 3′ and 5′ datasets were independently normalized to the expression of the top 96 housekeeping (HK) genes as identified by Lin et al. (Lin et al., 2019). For each cell, the average log-normalized expression of these genes was used to define a more stable “reference gene”. Expression values were then Z-transformed using the mean and standard deviation of this reference.

### 2.10 scVI and scArches adaptation

In addition to the standard methods described above, we adapted scVI (v1.3.1) (Lopez et al., 2018) for correcting 3′ vs. 5′ data. Assay information was provided as a batch key during model training with no other covariates. Training was performed on the entire set of genes, and hyperparameters were set based on the developer’s recommendations. Normalized (decoded) gene expression for 3′ cells was then obtained using “get_normalized_expression” with *transform_batch* = 5′ assay, and with 10,000 as the library size.

Using scArches (v0.6.1) (Lotfollahi et al., 2022), we projected 3′ scRNA-seq data onto 5′ scRNA-seq space. We used the scVI autoencoder model basis (Lopez et al., 2018) and adapted the existing batch projection method to project 3′ into the latent space of 5′. Unlike scVI, we trained scArches using a leave-one-out cross-validation strategy where one entire dataset was excluded from training and used to validate model performance. Normalized (decoded) gene expression was then obtained using “get_normalized_expression” with *transform_batch* = 5′ assay, and with 10,000 as the library size. This reconstructed 5′ scRNA-seq data was compared to the matching real 5′ scRNA-seq data.

### 2.11 Statistical performance measures

Since matched datasets were derived from the same tissue and subject but did not contain identical cells, we calculated the mean expression of every gene for each cell type within each dataset both before and after normalization. For each cell type, we computed Pearson correlation coefficient, cosine similarity, mean squared error (MSE), Euclidean distance, and Jensen-Shannon divergence (JSD) (philentropy v0.9.0) (Drost, 2018). Performance was assessed using the percent change for Euclidean distance and MSE. For bounded metrics, such as Pearson correlation, cosine similarity, and JSD, performance was assessed as percent change relative to the maximum change possible.

#### 2.11.1 Controls

As M3Drop and SCTransform rescale each gene to a mean of zero and a variance of one, they were compared to the combined 3′ and 5′ data after log-normalization and scaling. All other normalization methods were compared to log-normalized expression.

### 2.12 Comparing performance - percent and relative percent change

To compare the statistical performance of all correction methods, we calculated the percent change in each metric relative to the scores for the appropriate control (see Methods-Controls above). For bounded measures, we adapted the percent change calculated to account for the total possible improvement:

Bounded [-1,1] such as Correlation Coefficient and Cosine Similarity:

$$Relative\;Percent\;Change=\left\{\begin{array}{c} \frac{value-baseline}{1-baseline}*100,if\;value>baseline\\ \frac{value-baseline}{baseline+1}*100,if\;value<baseline \end{array}\right.$$

Bounded [0,1] such as JSD:

$$Relative\;Percent\;Change=\left\{\begin{array}{c} \frac{value-baseline}{1-baseline}*100,if\;value>baseline\\ \frac{value-baseline}{baseline}*100,if\;value<baseline \end{array}\right.$$

Unbounded such as Euclidean Distance and MSE.

$$Percent\;Change=\frac{value-baseline}{baseline}*100$$

### 2.13 UMAPs and mixing metric

Corrected 3′ and 5′ expression matrices were merged and processed using the standard Seurat v5 workflow (Hao et al., 2024). HVGs were obtained as described in “Data Preprocessing & Highly Variable Genes” section above. For methods other than M3Drop and SCTransform the data were scaled such that each gene had a mean of 0 and standard deviation of 1. Scaled corrected expression values were subjected to singular value decomposition for dimensionality reduction using Seurat (v5.1.0) to generate 40 principal components. Based on the proportion of variance explained by each component, the top 20 principal components were selected for downstream analysis. UMAPs were generated using default parameters, and the MixingMetric was calculated using Seurat. The neighborhood size was set to 150 cells to balance local and global structure.

### 2.14 Marker-gene overlap

Cell-type-specific markers were identified within corrected 3′ and corrected 5′ scRNA-seq datasets independently using a Wilcoxon rank-sum test. Significance was determined as 5% FDR. Marker consistency was defined as the intersection of 3′ and 5′ marker genes divided by the minimum number of significant markers in 3′ or 5′. These Overlap Scores were averaged across subjects for each cell type. Uncorrected log-normalized counts were used as a reference.

### 2.15 Biological use-case construction

We selected three of the matching 3′ and 5′ datasets (Park thymus (Park et al., 2020), Suo liver (Suo et al., 2022), Jardine bone marrow (Jardine et al., 2021)) to generate synthetic use-case scenarios and evaluate top-performing correction methods, as these datasets contained similar yet distinguishable cell types that were also sufficiently abundant within each subject (Supplementary Table S1). For each dataset, sparsity was introduced by subsetting 3′ and 5′ data such that a target abundant cell type was unique to each assay type (Supplementary Table S7). DEGs were identified between the two target cell types after correction and compared with the ground truth DEGs (outlined below). Datasets with 100% overlap (original samples) were used as controls.

The ground truth DEGs were defined for each dataset by first comparing the two selected cell types separately in the 3′ data and in the matched 5′ data (shown in Figure 4A). Genes were retained only when they were significant and directionally concordant in both assays, and the final dataset-level ground truth set was obtained by intersecting these concordant genes across all subjects. Significance was determined as 5% FDR. To account for limited power, we defined true positives (TP), false positives (FP), true negatives (TN), and false negatives (FN) as follows (shown in Figure 4B): TPs were significant in the ground truth and in the corrected comparison and were upregulated in the same cell type; FPs were any genes significant in both ground truth and in the corrected comparison but upregulated in the opposite cell types; FNs were genes upregulated in the same cell type and significant in the ground truth but not significant in the corrected values; TNs were genes that were not significant in either the ground truth or the corrected values, regardless of direction. These definitions were used to calculate Matthews correlation coefficient (MCC). We also calculated the total number of significantly up- and downregulated genes identified by each method at 5% FDR.

### 2.16 Computational demands

Runtime and memory usage were evaluated using the Park dataset which comprised nine subjects with an average of 4,415 cells per subject (total 39,709). Each correction method was run on a Digital Research Alliance of Canada (alliancecan.ca) high performance computing cluster node (Rorqual) with two x AMD EPYC 9654 (Zen 4) @ 2.40 GHz, 384MB cache L3. An exception was made for scVI, scArches, and Scanorama which were run on a personal computer with an Apple M3 Pro chip.

## 3 Results

### 3.1 Exploration of protocol-biased genes between 10X genomics 3′ and 5′ across 36 matched samples

We performed differential gene expression analysis on 36 subjects with matched 10X 3′ and 5′ scRNA-seq data, drawn from six publicly available datasets spanning multiple tissues (Supplementary Table S1) (Park et al., 2020; Jardine et al., 2021; Salcher et al., 2022; Suo et al., 2022; Andrews et al., 2024; De Simone et al., 2025). 3′ and 5′ data were compared for each subject separately to avoid any confounding by inter-subject differences. We identified between 609 and 2,598 genes consistently biased between platforms at different significance thresholds (1e^−5^, 1e^−50^, and 1e^−100^) (Figure 1A).

**FIGURE 1:**
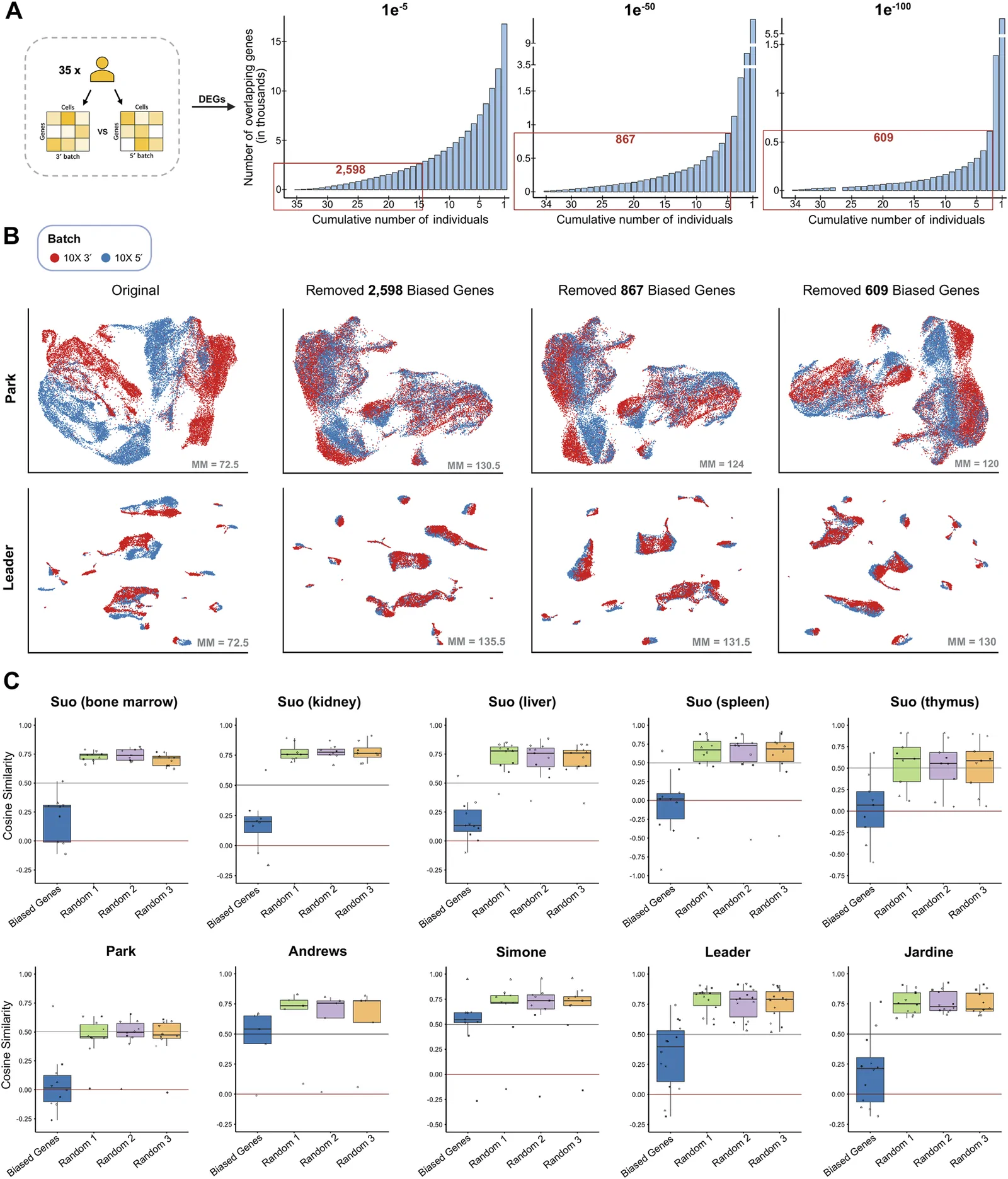
Exploration of differentially expressed genes (DEGs) between 10X 3′ and 10X 5′ protocols across 36 matched samples. Preprocessed matched 3′ / 5′ scRNA-seq samples from 36 subjects across six datasets spanning multiple tissues were obtained from the CELLxGENE platform and analyzed in R (v4.4.1) using Seurat (v5.1.0). **(A)** Cumulative distribution of overlapping DEGs across subjects at three significance thresholds (1e^−5^, 1e^−50^, 1e^−100^). Bars indicate the number of shared DEGs as more subjects are considered cumulatively; red labels show the total DEGs chosen at each threshold for further investigation. **(B)** UMAP visualizations from representative datasets (Park and Leader) showing cells from 10X 3′ (red) and 10X 5′ (blue) protocols. The first column shows the original data, while subsequent columns display cell distributions after removal of biased genes (DEGs corresponding to the chosen number in red from Panel A). For each plot, the value of the Seurat MixingMetric is indicated in gray at the bottom right corner, with higher values representing better batch mixing. **(C)** Boxplots of cosine similarity between average gene expression profiles of 3′ and 5′ data, comparing scores for the 867-gene list (1e^−50^ threshold) to three replicates of randomly selected gene sets of equal size and expression proportions across five representative datasets. Cell types are shown as different shapes. Horizontal lines at 0 and 0.5 are shown to aid comparison across datasets.

Removing each of the three biased gene sets improved the alignment between matched 3′ and 5′ data in the lower-dimensional space (Figure 1B). UMAPs showed clear assay-associated separation in the original data that was largely reduced after the biased genes were excluded, while cell-type concordance was improved (Figure 1B; Supplementary Figures S2, S3). In addition, the MixingMetric (Stuart et al., 2019) showed a striking increase upon removing as few as 609 genes, with a smaller gain as larger gene sets were removed. At the expression level, the 609- and 867-gene biased sets showed substantially lower cosine similarity between matched 3′ and 5′ cell-type profiles than random gene sets matched in number and expression composition (0.25 vs. 0.5–0.75, respectively), indicating that biased genes captured a disproportionate share of the protocol-associated expression differences (Figure 1C; Supplementary Figure S4). We therefore selected the 867-gene set, hereafter referred to as the *biased genes*, for downstream analysis as it provided a balanced compromise - smaller gene sets resulted in lower alignment of 3′ and 5′ data in several datasets, while larger sets offered little additional improvement while removing substantially more genes (Figure 1B; Supplementary Figure S2).

To test whether this biased-gene set generalized beyond the samples used for discovery, we evaluated the 867-gene list in seven additional matched 3′/5′ validation comparisons that were not used to define the gene set. These validation subjects differed in assay composition and cell-type representation but showed similar protocol-associated structure. Removal of the predefined 867 genes improved 3′/5′ mixing in UMAP space in all validation comparisons, as reflected by increased Seurat MixingMetric values. Consistent with the discovery analysis, the biased-gene set showed lower 3′/5′ cosine similarity than expression-matched random gene sets, indicating that these genes captured recurrent protocol-associated expression differences in held-out matched data. Additionally, the number of significantly differentially expressed genes among the biased gene list (FDR <0.05) matched between the validation subjects and discovery subjects, when accounting for the total number of cells (Supplementary Figure S5).

To assess whether the identified biased genes were comprehensive, we examined their recurrence across subjects, as well as their expression patterns and functional properties. The total number of genes significant at FDR <10^–50^ varied across subjects, where those with the most cells generally had the largest number of significant genes (Supplementary Figure S6A). Thus, the final biased gene set was selected based on recurrence across subjects, ensuring that the list was not dominated by highly powered subjects. Consistent with this, most subjects showed significant 3′/5′ differences for at least 200 of the 867 genes, although no individual subject contained the full set (Supplementary Figure S6B). Conversely, most genes in the final list were significant in more than nine subjects (Supplementary Figure S6C).

Across the six datasets, 44% of all human genes and 75% of all protein-coding genes were detectable, defined as being expressed in more than 1% of cells in at least one dataset (Supplementary Figure S7Ai, ii). The undetected protein-coding genes were enriched for tissue-specific genes associated with major tissues not represented in our datasets, particularly the brain and reproductive organs, as evidenced by the enrichment of neuronal, sensory, developmental, and reproductive pathways (Supplementary Figure S7Aiii). Protocol-biased genes tended to have high expression across multiple datasets, with 75% in the highest expression quantile in most datasets (Supplementary Figure S7B). These genes were also enriched for housekeeping functions, including cellular translation and oxidative phosphorylation (Supplementary Figure S7C). Although the majority of chosen datasets were derived from immune-rich tissues, as 5′ scRNA-seq is predominantly used to study T and B cell clonality, nine protocol-biased genes were specific to a non-immune cell type. This finding indicates that our analysis had the power to detect protocol-biased genes across a broad range of tissues and cell types (Supplementary Figure S7D).

While we observed the known trend of higher GC content at the 5′ end of transcripts compared with the 3′ end, biased genes showed no differences in this GC-content pattern, suggesting that the protocol differences are not primarily driven by PCR amplification biases (Aird et al., 2011) against GC-rich sequences (Supplementary Figure S7E). Additionally, biased genes were slightly shorter than expected, although this is likely explained by their enrichment for housekeeping functions, as it is known that highly expressed housekeeping genes tend to be shorter (Eisenberg and Levanon, 2003). Since first exons are generally shorter than last exons, 5′ -end capture may be more sensitive to exon-only quantification if a larger fraction of reads from the 5′ end map near exon–intron boundaries or to unspliced transcript regions (Movassat et al., 2019). As nine of ten datasets were generated using pipelines in which only exonic reads were counted when calculating UMIs per gene per cell by default (Supplementary Table S1), differences in exonic vs. intronic composition near the 5′ and 3′ ends of transcripts could therefore contribute to some protocol-associated differences. We observed that most protocol-biased genes had higher expression in 5′ compared with 3′ samples, and 39 biased genes had no annotated introns, indicating that exon-only counting might be insufficient to explain the protocol-biased genes (Supplementary Figure S6D). Additionally, to test this directly, we remapped one matched Suo spleen subject using exonic-only and exonic-plus-intronic quantification. Including intronic reads reduced the number of significant genes from the predefined 867-gene set from 525 to 390, primarily by reducing the number of significant genes with higher expression in 5′ libraries. However, most biased genes remained significant, indicating that intronic-read inclusion attenuates a subset of biased-gene calls but is insufficient to explain all the recurrent 3′ / 5′ protocol bias (Supplementary Figure S6E-G).

These results indicate that a relatively small subset of genes accounts for most of the expression differences between 10X 3′ and 5′ chemistries in the evaluated datasets, while the remainder of the transcriptome appears largely consistent across protocols.

### 3.2 Benchmarking of eleven common scRNA-seq batch correction methods for mitigating 10X 3′ / 5′ protocol bias

We compared the performance of eleven well-known normalization methods (Supplementary Table S6) (Johnson et al., 2007; Ritchie et al., 2015; Haghverdi et al., 2018; Lopez et al., 2018; Andrews and Hemberg, 2019b; Hafemeister and Satija, 2019; Lotfollahi et al., 2022) in their ability to correct the gene-expression values of 3′ and 5′ scRNA-seq data while preserving the biological variation (Figure 2A). To mitigate confounding effects from inter-subject genetic heterogeneity, all normalization methods were applied independently to the matching 3′ and 5′ samples for each of the 36 subjects. Samples with substantial differences in cell-type frequency between 3′ and 5′ data were excluded to avoid biases from incomplete dissociation.

**FIGURE 2:**
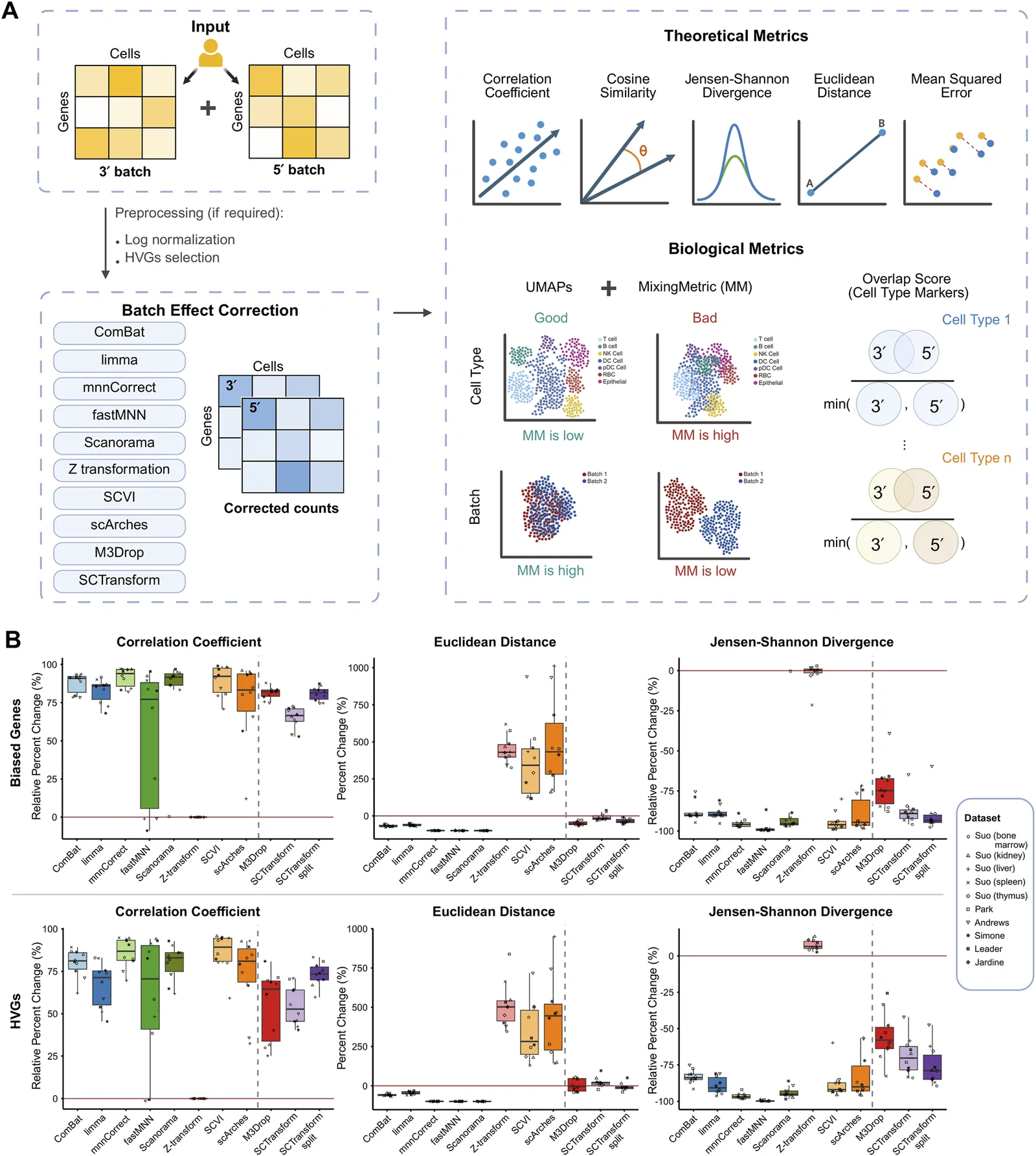
Benchmarking of eleven common scRNA-seq batch correction methods for mitigating 10X 3′ / 5′ protocol bias. Matched samples from 36 subjects across six datasets were independently normalized using each technique. **(A)** Benchmarking workflow. **(B)** Boxplot of three representative metrics comparing the eleven correction techniques across all datasets (shown as different shapes), with zero (red line) indicating the respective baseline. The dashed gray line indicates that the baseline used for ComBat through scArches is different from that of M3Drop and SCTransform (log-normalized vs. log-normalized and scaled counts respectively). Higher correlation coefficients and lower values for other metrics denote better integration performance. Correlation coefficient and JSD results are shown as relative percent change; Euclidean distance results are shown as percent change. The top panel shows the metrics for biased genes from Figure 1, and the bottom panel shows the metrics for the 3,000 Highly Variable Genes. Created in BioRender. Shydlouskaya, V. (2026) https://BioRender.com/lktft3q.

### 3.3 Quantitative measures of corrected 3′ vs. 5′ similarity

Performance was assessed by comparing cell-type-specific expression of the previously identified biased genes and 3,000 highly variable genes (HVGs) between the corrected 3′ and 5′ scRNA-seq data relative to an appropriate reference (Figure 2B; Supplementary Figure S8). ComBat (Johnson et al., 2007) and mnnCorrect (Haghverdi et al., 2018) consistently yielded the largest improvements over the uncorrected baseline by increasing the Pearson correlation between 3′ and 5′ expression and reducing JSD and Euclidean distance. In contrast, our housekeeping-gene-based Z-transformation performed the worst with no evidence of improving 3′ and 5′ concordance.

Other methods had differing results depending on the metric used. FastMNN (Haghverdi et al., 2018) performed similarly using Euclidean distance and JSD but had variable performance using Pearson correlation. Limma (Ritchie et al., 2015) performed similarly to ComBat except for weaker performance on the correlation. Scanorama (Hie et al., 2024) also improved Pearson correlation, cosine similarity, and JSD relative to the uncorrected baseline, but it did not consistently outperform mnnCorrect or fastMNN and showed weaker performance for some distance-based measures. The machine-learning approaches, scVI (Lopez et al., 2018), and scArches (Lotfollahi et al., 2022), were comparable in performance to the others using the correlation and JSD but substantially increased the Euclidean distance between 3′ and 5′ vs. baseline. Results using cosine similarity and MSE were consistent with those for Pearson correlation and Euclidean distance respectively (Supplementary Figure S8).

In contrast to the other methods, the Pearson-residual-based approaches, M3Drop (Andrews and Hemberg, 2019b) and SCTransform (Hafemeister and Satija, 2019), showed substantially better performance on the biased gene set than on the HVGs, particularly when considering the Euclidean distance and MSE (Supplementary Figure S8A). Somewhat surprisingly, SCTransform run using its “split” option, which fits regression independently for each sample, substantially outperformed the regression-model approach used to correct for 3′ vs. 5′ differences across all metrics and gene sets.

### 3.4 Biological measures of correction performance

We assessed the performance of each normalization method to align 3′ and 5′ datasets in lower-dimensional space (Figure 3A). Performance was quantified using the MixingMetric for 3′ and 5′ labels (Figure 3B). The top-performing methods were ComBat, fastMNN, and M3Drop, which produced complete overlap of 5′ and 3′ scRNA-seq datasets without disrupting cell-type clustering. Scanorama showed variable performance when UMAPs were generated from its corrected cell-by-gene expression matrix. In contrast, UMAPs generated from Scanorama embeddings showed stronger 3′ / 5′ alignment in several datasets while preserving broad cell-type structure (Supplementary Figure S9). This distinction suggests that Scanorama performs better as a latent-space integration method than as a source of corrected expression values for downstream gene-level analyses. Meanwhile, limma, Z-transformation, and SCTransform regression failed to integrate 3′ and 5′ datasets. Consistent with our observations above, the “split” approach to SCTransform consistently outperformed the regression model.

**FIGURE 3:**
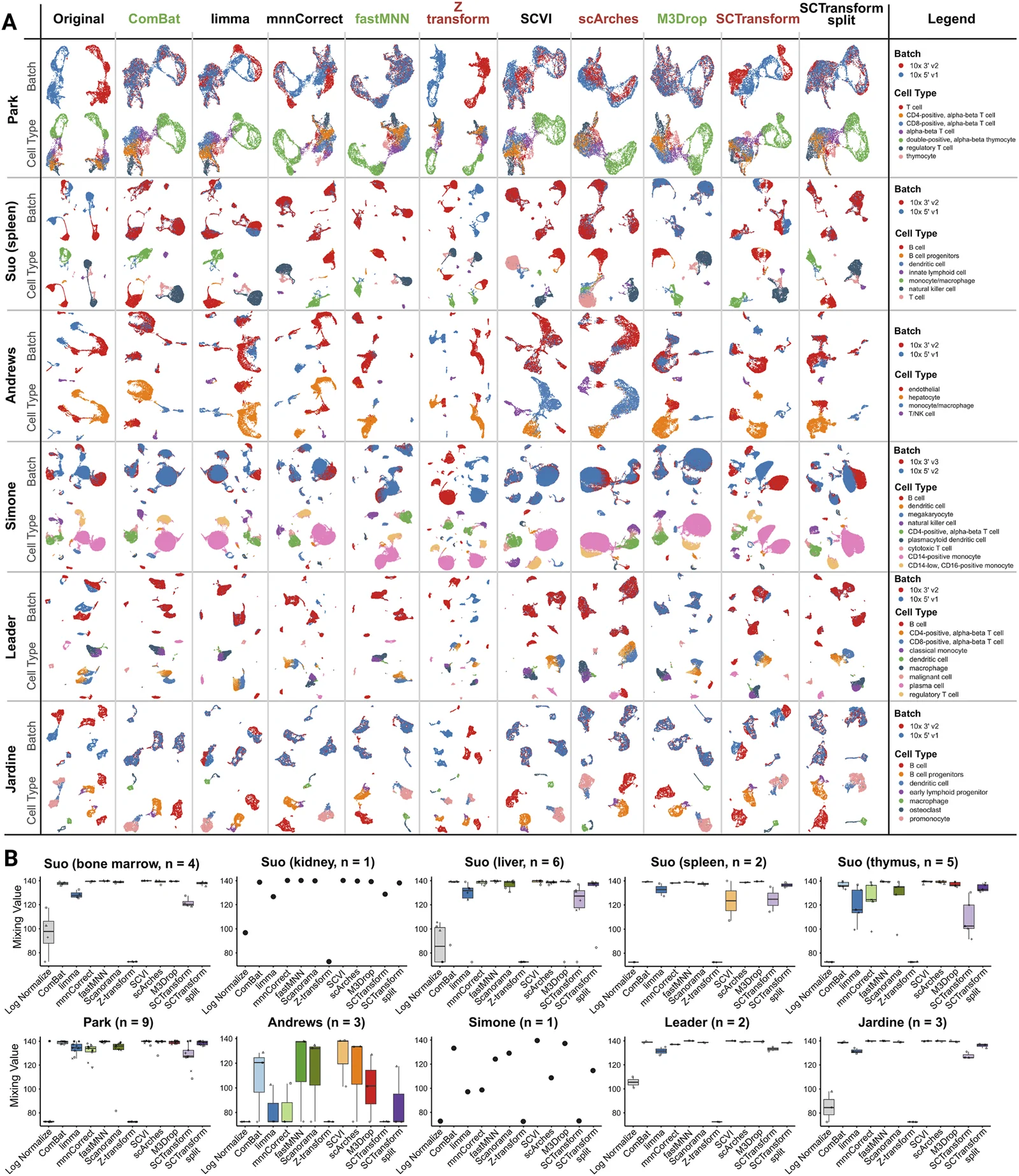
Evaluation of eleven common scRNA-seq batch correction methods for preserving cell type structure after 10X 3′ / 5′ correction. Matched samples from 36 subjects across six datasets were independently integrated using each technique based on the workflow shown in Figure 2A. **(A)** UMAP visualizations following different batch correction methods, using log-normalized counts as a reference. Cells are colored by sequencing protocol (top) and by cell type (bottom–as identified by the authors). Only representative plots of datasets, with one subject per dataset, are shown. Best- and worst-performing method names are indicated in green and red, respectively. **(B)** Boxplots showing Seurat MixingMetric results by batch (assay) for all datasets. Shapes represent different subjects. Higher values indicate better mixing of cells across batches.

All methods were able to preserve cell-type distinctions, with a MixingMetric of 0 for nine of the ten datasets, when evaluated using cell-type labels. In the remaining dataset, Z-transformation and scArches performed worse than the other correction methods in preserving cell-type identities (Supplementary Figure S10).

We also assessed the consistency of cell-type-specific marker genes identified between 3′ and 5′ data (Supplementary Figure S11). While fastMNN, scVI and scArches achieved the highest marker overlap, this observation was likely driven by an inflated number of significant marker genes, by 6–10-fold, reaching 7,000-30,000 for some cell types. Meanwhile, Scanorama consistently yielded fewer intersecting 3′ / 5′ marker genes than any other method but showed variable performance for marker overlap across datasets. This indicates that Scanorama did not drive the same broad marker-gene inflation observed for some other integration methods, but conversely, its correction may reduce both noisy and biologically meaningful signals between the cell types, leading to weaker recovery of cell-type marker genes.

Interestingly, removing the 867 biased genes had negligible effects on identification of consistent cell-type-specific marker genes, while substantially reducing the batch effect in the lower-dimensional space (Supplementary Figures S2, S3, S11). Thus, despite improvements in 3′ and 5′ expression concordance, it is unclear whether correction improves the biological interpretation of the data.

### 3.5 Computational cost of correcting 3′ and 5′ gene expression

Most normalization methods scaled well, requiring <20 GB of RAM, and completed in <5 min of runtime on two x AMD EPYC 9654 (Zen 4) CPUs at 2.40 GHz, 384MB cache L3. The exception was mnnCorrect which required 30.55 GB of RAM and 51 min of runtime for the Park dataset (Park et al., 2020) comprising nine subjects with an average of 4,415 cells per subject and ∼40,000 cells total (Supplementary Table S6). As expected, deep learning methods, scVI and scArches, required longer runtimes but were run on a different hardware, Apple M3 Pro chip.

### 3.6 Applying batch correction methods to integrate partially overlapping 3′ and 5′ datasets

Finally, we simulated a real-world application scenario in which we corrected datasets with incomplete cell-type overlap between 3′ and 5′ samples. We introduced artificial sparsity into three selected datasets of different tissues (thymus, liver, and bone marrow) such that only ∼25% of cells were shared among 3′ and 5′ samples, with some cell types left to be unique to each batch (Figure 4A). As an easier test, we considered a scenario with complete (100%) overlap. We focused our evaluation on the well-performing normalization methods identified in earlier analyses, assessing their ability to recover correct cell-type markers between two selected populations. Specifically, we tested limma, fastMNN, Scanorama, scVI, and M3Drop, along with log-normalized data with and without removal of the 867 protocol-biased genes. Limma was chosen in place of ComBat, as an algorithmically similar linear method, since ComBat does not handle large cell-type discrepancies between integrated datasets well (Nygaard et al., 2016; Haghverdi et al., 2018).

**FIGURE 4:**
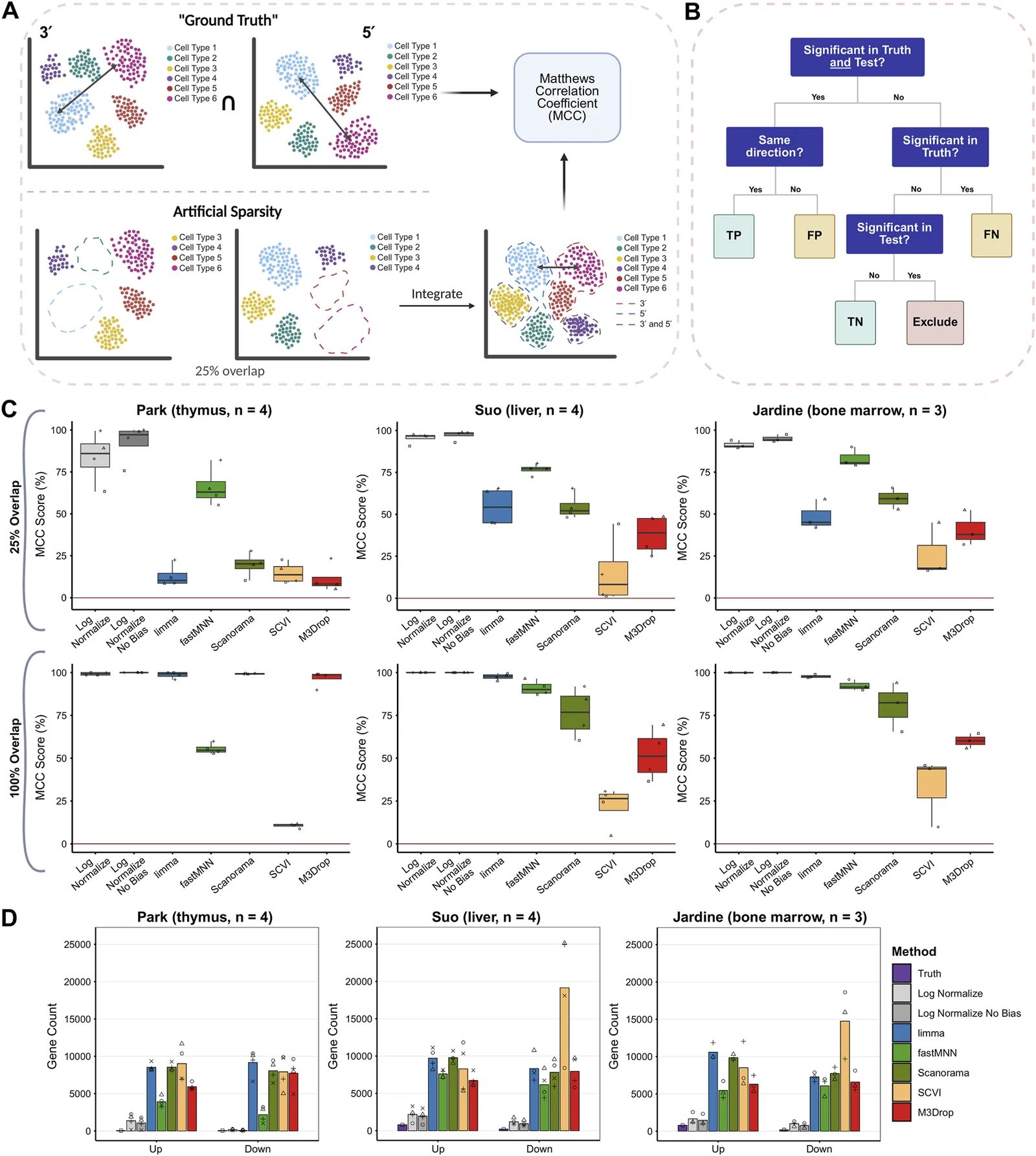
Evaluation of selected batch correction methods in their ability to recover correct cell type markers. For each subject, selected batch correction approaches (limma, fastMNN, Scanorama, scVI, and M3Drop) were evaluated for their ability to recover cell-type-specific differential expression after 3′ / 5′ library correction in three selected datasets: Park (n = 4 subjects), Suo liver (n = 4 subjects), and Jardine (n = 3 subjects). Log normalization was used as an uncorrected reference. **(A)** The ground truth DEG set was defined as the intersection of genes differentially expressed between the two chosen cell types separately within the 3′ and 5′ data, followed by their intersection. The resulting DEG sets were then intersected across subjects to define a single ground-truth set. In each subject, cells were subset such that common cell types between 3′ and 5′ datasets accounted only for 25% of all cells. Finally, subset datasets were corrected, and performance was quantified using the Matthews correlation coefficient (MCC). **(B)** MCC was calculated based on custom TP/FP/FN/TN definitions. **(C)** Boxplots show the distribution of MCC scores across subjects (represented with shapes) for each batch correction method and dataset; higher MCC indicates greater agreement with the ground truth. **(D)** Bar plots show the number of significantly up- or downregulated DE genes between selected cell types after correction. Created in BioRender. Shydlouskaya, V. (2026) https://BioRender.com/ao36n4e.

To evaluate performance in these scenarios, we selected 2 cell types with high frequency in both 3′ and 5′ datasets for all associated subjects and moderate biological distinctiveness. Ground truth differentially expressed (DE) genes between these 2 cell types were identified using a Wilcoxon rank-sum test in 3′ and in 5′ data independently. Those genes consistently DE in all subjects in both 3′ and 5′ were considered truly DE between those cell types (Figure 4A; Supplementary Figure S12). Cells from one target cell type were removed from the 3′ sample, and cells from the other target cell type were removed from the 5′ sample, and the datasets corrected using each selected approach, as well as by excluding the protocol-biased genes we identified (Figure 4A). Performance was assessed by comparing DE genes between the cell-types after correction to the ground truth genes using the Matthews correlation coefficient (MCC). To account for possible power differences, MCC was calculated using a power-agnostic classification (Figure 4B). Across both the sparse- (25% overlap) and full-overlap settings, log-normalized data generated after removing the 867 biased genes produced the highest MCC scores, followed by uncorrected log-normalized expression (Figure 4C). These results suggest that, in this context, batch correction may not improve, and may even impair, the accuracy of marker gene detection. Among correction methods, fastMNN stood out for its consistent performance even in the 25% overlap scenario, achieving the next highest MCC scores, however still lower than simple log-normalization. In addition, unlike many other correction techniques, fastMNN only slightly increased the total number of DE genes, indicating good control of false positives (Figure 4D). Scanorama and limma performed similarly in the 25% overlap setting and inflated the DEG counts, although the strongest inflation was observed for scVI (Figures 4C,D).

The poor performance of the correction methods in this setting was not attributable to the selected ground-truth marker genes lacking differences between 3′ and 5′ assays, as in all three cases at least 10% of the ground truth markers overlapped with our biased gene set, which by definition contains genes with large 3′ / 5′ expression differences between 3′ and 5′ assays (Figure 5A). Indeed, inspection of some classic cell-type markers for our chosen cell types, which were found among the ground truth genes, showed clear differences between 3′ and 5′ (Figure 5B).

**FIGURE 5:**
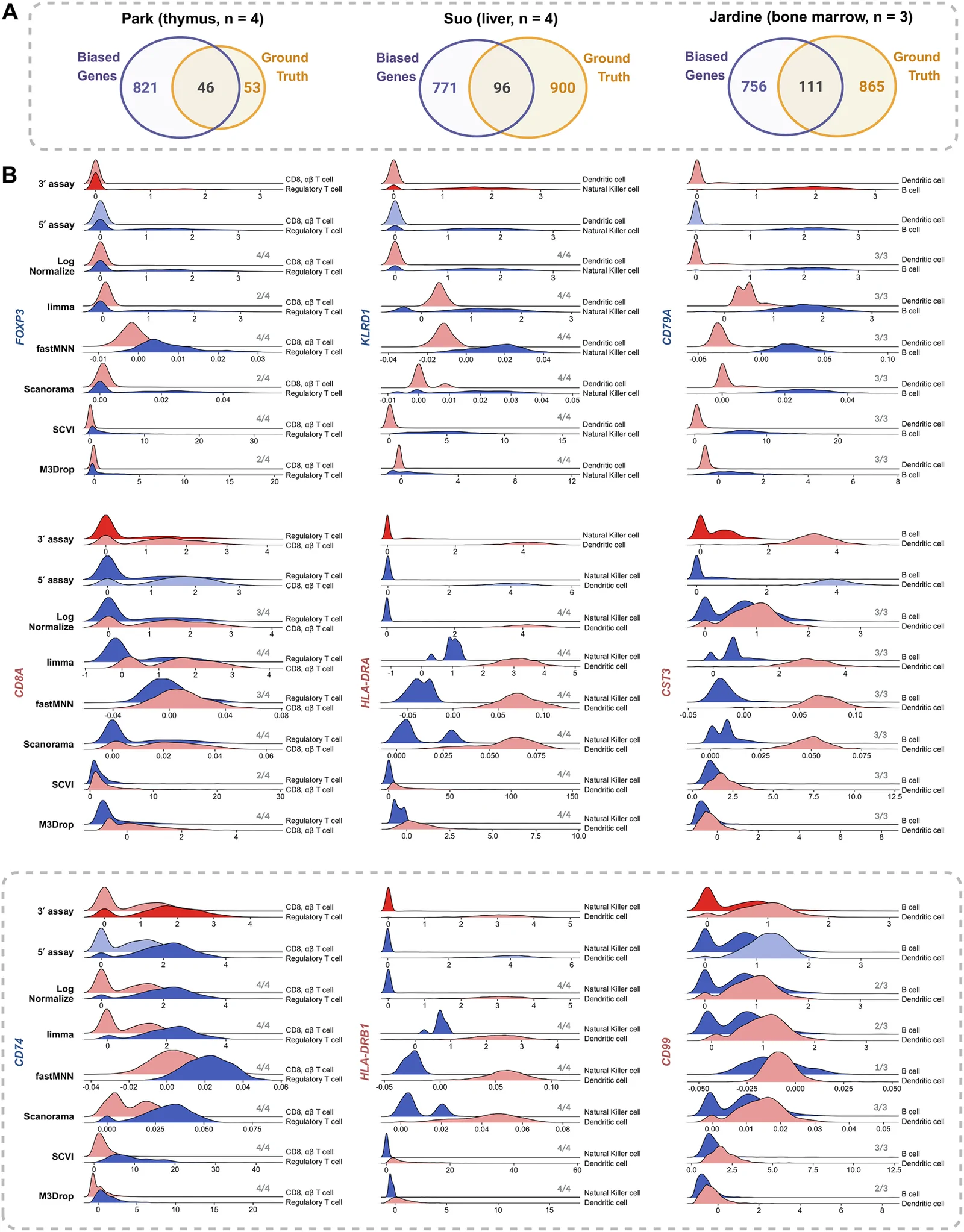
Evaluation of cell marker gene expression for selected batch correction methods. Park (n = 4 subjects), Suo liver (n = 4 subjects), and Jardine (n = 3 subjects) datasets were preprocessed as described in Figure 4 (Panel A). **(A)** Overlap between protocol-biased genes and the ground truth cell-type marker genes for the selected datasets. Numbers indicate the absolute number of DE genes in the intersection or unique to each set. **(B)** Ridge plots illustrating the gene expression distributions for representative marker and protocol-biased genes after different normalization methods. For each dataset, the first two gene groups show true marker genes, one for each of the 2 cell types being compared. Within each gene group, the first two rows show expression from the uncorrected 3′ (red) and 5′ (blue) assays, using log-normalized counts. Subsequent rows show the same genes after normalization with each top-performing method. The final gene group (outlined with a gray dashed box) displays an example gene that was both a ground truth cell-type marker and among the 867 biased genes. The gene name color represents which cell type the gene is the canonical marker for (red–cell type retained in 3′ ; blue–in 5′ ). The gray numbers for each ridge plot show the number of subjects that had this gene in their marker gene list for the respective cell type after correction.

While correction methods did substantially alter the expression of these genes, they failed to accurately remove the specific bias introduced by 3′ vs. 5′ technologies. Notably, fastMNN and scVI generally smoothed and imputed expression values in addition to correcting them, which successfully preserved DE patterns for genes with clear differences, but which sometimes led to over-smoothing in genes with more overlapping distributions such as CD99 (Figure 5). fastMNN and Scanorama also uniquely compressed the range of gene-expression values, with corrected values typically falling within the [0, 0.1] range. Meanwhile, limma and M3Drop tended to alter zero values in a manner that contradicted the expected DE pattern, leading to a loss of significance in genes such as FOXP3 and KLRD1. Notably, only limma and Scanorama more frequently preserved bimodality of expression when present, as observed for CST3 and CD99 genes. Additionally, Scanorama largely retained the near-zero expression intact rather than broadly shifting low-expression cells to negative values. However, this did not consistently translate into improved biological accuracy, as Scanorama sometimes introduced abnormal distributional shifts in the expressing cell population, as observed for KLRD1.

These findings highlight the challenge of correcting protocol-specific effects without compromising biological variation. Although 10X 3′ and 5′ protocols introduce detectable expression biases, these differences are largely confined to a small subset of genes. Across matched samples from diverse tissues, most batch correction methods performed comparably on statistical benchmarks, with fastMNN, mnnCorrect, and SCTransform (split) showing consistently strong alignment of expression profiles. However, when evaluated in a biologically relevant context, the performance shifted. Methods such as fastMNN maintained robust marker recovery, while others that performed relatively well in statistical metrics, such as scVI or M3Drop, showed inflated gene detection or distorted expression dynamics. Importantly, we found that excluding the small set of 867 protocol-biased genes prior to analysis substantially improved downstream alignment and may provide a more reliable alternative to complex correction workflows in certain scenarios.

## 4 Discussion

Both 3′ and 5′ scRNA-seq chemistries are widely used, with the 5′ protocol commonly employed for immune repertoire analysis due to its ability to capture recombined TCR and BCR sequences (Redmond et al., 2016; Pai and Satpathy, 2021), while the 3′ protocol remains prevalent in large single-cell atlases due to its cost efficiency and historical use (Han et al., 2018; Cao et al., 2019; 2020; The Tabula Sapiens Consortium, 2022). As a result, publicly available scRNA-seq datasets increasingly comprise a mixture of 3′ and 5′ chemistries, creating both opportunities and challenges for cross-protocol comparisons and meta-analyses. Most existing batch correction methods only align datasets in a reduced-dimensional space, rather than correcting gene-level expression values directly (Tran et al., 2020; Luecken et al., 2022). We therefore focused on methods that directly return corrected gene expression values or can be adapted to generate gene-level corrected or reconstructed outputs and that do not require cell type label information. Clear guidance on whether, and how, gene expression values from 3′ to 5′ protocols can be meaningfully compared is lacking. Our study addresses this gap by characterizing protocol-specific expression differences and evaluating normalization and correction strategies in biologically realistic settings.

Interestingly, most protocol-induced variation was driven by a relatively small subset of genes (Figure 1). Such variation is thought to arise from transcript-specific differences in capture and amplification efficiency (Yamawaki et al., 2021; Hsu et al., 2022). However, the biased genes were predominantly upregulated in 5′ relative to 3′ data and showed no greater disparity in GC content between their 5′ and 3′ ends than unbiased genes, providing little support for GC-dependent amplification bias as the primary explanation (Supplementary Figures S6, S7). Intronic versus exonic quantification also did not fully explain the observed protocol-biased genes. The RG1237 sample from De Simone et al. (2025), which was processed using a Cell Ranger version that includes intronic reads by default, still showed substantial recurrence and 5′ -skewed directionality of the biased genes. Moreover, after remapping one Suo spleen sample with and without intronic reads, we find that 25% (135/525) significant biased-gene calls were sensitive to intronic-read inclusion in that sample (Supplementary Figures S6Gii). However, the 5′ -skewed directionality persisted. Thus, intronic-read inclusion is insufficient to explain most of the recurrent protocol-associated bias. We can only speculate that other gene-level features, such as local sequence composition, transcript structure, or sequence homology, may influence transcript capture rates in the different technologies and contribute to the remaining unexplained bias.

Since we observe the identities of the biased genes and the direction of their bias to be highly conserved across datasets, we expect the bias originates from an independent technical effect. However, since the 10X chemistry for 3′ vs. 5′ is comparable, and use the same reagents, except for transcript-end capture and barcode/UMI incorporation design (10X Genomics, 2025) it is unlikely to be a result of the specific protocol being used but rather a characteristic of the genes themselves that creates the bias. Thus, we expect the bias to be conserved across other single-cell RNA-seq platforms, such as BD Rhapsody, Drop-seq, or Seq-Well, but is less likely to be conserved between species.

We observed that the identified protocol-biased genes tended to be highly expressed and broadly distributed across tissues. Additional validation in held-out matched 3′ / 5′ comparisons supported recurrence of the biased-gene signal beyond the samples used for discovery. However, the diversity of tissues included in our analysis also enabled the detection of protocol-biased genes with lower expression and those specific to non-immune cell types (Supplementary Figure S7). Since 5′ scRNA-seq is typically applied to immunologically complex tissues, the biased genes identified here are likely to be broadly applicable to the tissue types for which paired 5′ and 3′ scRNA-seq datasets are available or may be generated.

### 4.1 Performance of gene-level batch correction methods across statistical benchmarks

Across multiple statistical metrics and datasets, fastMNN, mnnCorrect, and ComBat consistently improved agreement between matched 3′ and 5′ expression profiles as measured by statistical similarity (Figure 2). These findings align with previous benchmarking studies showing that mutual nearest neighbor-based approaches and linear batch correction methods perform robustly in settings with moderate batch effects and shared cell populations (Haghverdi et al., 2018; Luecken and Theis, 2019; Tran et al., 2020). While mnnCorrect often achieved slightly stronger alignment than fastMNN, it did so at a substantially higher computational cost, both in memory usage and runtime, making it prohibitive for use in large-scale datasets or atlasing efforts. ComBat also performed well on statistical metrics, but its reliance on global linear adjustments limits its applicability in scenarios with strong cell-type imbalance or incomplete overlap between batches (Johnson et al., 2007; Tran et al., 2020). Notably, the Andrews dataset (Andrews et al., 2024), which had more divergent cell-type frequencies between 3′ and 5′ data, was the most difficult for the methods to correct, and only fastMNN and scVI were able to fully mix the two batches for this dataset (Figure 3).

Interestingly, we observed a consistent difference in the behavior of SCTransform variants (Figures 2, 3). Removing 3′ vs. 5′ differences using a negative binomial regression model consistently underperformed compared to applying SCTransform separately to the 3′ and 5′ data, without any explicit attempt to remove the differences between the assays. A likely explanation for this observation is that SCTransform fits a regression model to a subset of genes and then interpolates regression coefficients across genes with similar baseline expression levels. We showed that 3′ vs. 5′ differences are gene-specific and not consistent across genes of the same expression level, which would prevent SCTransform from accurately interpolating parameters of an appropriate correction. In contrast, when SCTransform is applied separately to each protocol, the difference in baseline expression levels of the protocol-biased genes is regressed to zero in each case, effectively eliminating the protocol bias.

Notably, some of our findings did not agree with previous integration benchmarks (Luecken et al., 2022; Tran et al., 2020). These other benchmarks evaluate performance through latent-space alignment, batch mixing, biological conservation, label transfer, and clustering. In contrast, our benchmark asks whether corrected or reconstructed gene expression values preserve gene-level concordance and downstream differential expression between matched 3′ and 5′ datasets. These goals are related but not equivalent; for example, Scanorama can return a corrected cell-by-gene matrix, but this output is generated through an alignment framework originally developed for dataset stitching. When we constructed a lower-dimensional space from the corrected gene expression values it did not match the integrated lower-dimensional space obtained directly from Scanorama embeddings and in several cases was noticeably worse (Supplementary Figure S9). This indicates significant challenges remain in the translation from lower-dimensional space to gene expression values when integrating scRNA-seq data.

It is likely a similar issue explains the poor performance of scVI, and the related scANVI, in our study compared to previous benchmarks. scVI performs preprocessing including normalization of gene expression values prior to training the VAE in order to increase compatibility with the Gaussian distribution used in the stochastic component of the VAE and to prevent the VAE from learning features that correspond to total UMIs per cell. As a result, reconstruction of gene expression values from scVI is more complex than simply generating output from the decoder. We utilized normalized output values from scVI in our benchmark, as when we attempted to translate back to raw UMI-counts performance was substantially worse. In addition, due to the variational aspect of the model there is a wide distribution of possible decoder output values for each individual cell input values and it is unclear how to translate that distribution into a single column of output expression for a given cell input.

For nearly a decade the focus of scRNA-seq data integration has been on the lower-dimensional space as in most cases the samples being integrated are biological replicates. Thus, the technical and biological noise are fundamentally confounded and impossible to separate; therefore, differential expression results after “correcting” these counts would be statistically invalid. In this manuscript, we focus specifically on the 5′ vs. 3′ batch effect using datasets with matched samples from the identical biological source as it provides the rare case when technical and biological noise can be cleanly separated, thus gene-expression value correction would be statistically valid. A consequence of this method-development focus is that many popular integration methods do not produce corrected gene expression values (Korsunsky et al., 2019; Polański et al., 2020; Stuart et al., 2019), and those that do rarely optimize the performance of their reconstructed expression.

### 4.2 Statistical alignment does not necessarily translate to biological accuracy

When we further evaluated the normalization methods in a biologically relevant setting, involving comparisons between cell types present only in 5′ datasets with those present exclusively in 3′ datasets, a different pattern emerged (Figures 4, 5). In this scenario, when the 867 biased genes were excluded, the log-normalized data consistently achieved the highest agreement in differential expression between chosen cell types. Among correction methods, fastMNN remained comparatively robust but still underperformed relative to the uncorrected baseline.

This result highlights an important distinction between improving global similarity metrics and preserving biologically meaningful differences. Methods that performed well on distance- or divergence-based measures often identified substantially larger DEG sets, inflating both true positives and false positives (Figure 4; Supplementary Figure S11). Despite controlling the number of DEGs well when there was complete overlap of cell-types, Scanorama inflated the number of DEGs in the partial-overlap case. This suggests for some methods performance can depend strongly on the structure of the data being corrected.

The machine-learning methods, scVI and scArches, were particularly prone to inflating the number of DEGs, as one of their explicit goals is to denoise the data - a process that has previously been shown to inflate false-positive DEGs (Andrews and Hemberg, 2019a; Hou et al., 2020) (Figure 4D). Our findings are consistent with prior observations that aggressive batch correction can obscure or distort differential expression signals, particularly when biological differences are subtle or partially confounded with batch structure (Nygaard et al., 2016; Tran et al., 2020).

### 4.3 Batch correction may be unnecessary for many 3′ / 5′ comparisons

Taken together, our results suggest that gene-level batch correction between 3′ and 5′ scRNA-seq data may be unnecessary, and potentially detrimental. This conclusion was further supported by validation analyses showing that removal of the fixed 867-gene set improved concordance in additional matched 3′ / 5′ comparisons not used for gene discovery. Given that protocol-related differences are contained within a small gene set, a simpler strategy of excluding these biased genes may be sufficient to enable meaningful gene expression comparisons across protocols.

While the sets of protocol-biased genes we provide in Supplementary Tables S8-S10 are broadly representative of typical tissues to which 5′ scRNA-seq is likely to be applied, they may not be exhaustive. Lowly expressed genes are underrepresented due to the statistical challenges of their high sparsity in scRNA-seq datasets. In addition, some tissues do not have matching 3′ and 5′ datasets available and thus are not represented in this list. Expanding this work to include additional tissues, especially brain and reproductive organs, would enable a more comprehensive identification of protocol-biased genes.

Furthermore, due to the design of this study we were able to control for many other sources of variation such as sequencing depth, tissue dissociation, and individual genetic differences, which would also confound attempts to compare expression between different published datasets. Similarly, we limit our analyses to intra-subject comparisons to isolate the effect of protocol bias rather than considering multi-subject between group comparisons, which are increasingly the focus of scRNA-seq studies (Crowell et al., 2020; Heumos et al., 2023; Lin et al., 2023; Nesari et al., 2026). Thus, it remains unclear whether statistically rigorous cross-dataset and/or multi-dataset comparisons are possible, which may limit the potential of ongoing atlasing efforts (Ryu et al., 2023).

Although our analysis focuses on differences between 10X Genomics 3′ and 5′ chemistries, our observations may extend to other closely related scRNA-seq technologies that differ only in enzyme formulations or RNA capture strategies, such as 10X v2 and v3. However, more substantially different technologies such as those using probe-based transcript quantification or different reaction volumes may have more pervasive and non-linear biases (Lafzi et al., 2018; Hatje et al., 2025; Sarieva et al., 2026). Prior work has shown that such technology-driven effects are often gene-specific and do not manifest as uniform global shifts across the transcriptome (Lake et al., 2017). Thus, aggressive batch correction may distort quantitative gene expression and should be applied cautiously when batch effects are modest and biology is shared (Luecken and Theis, 2019; Tran et al., 2020). Since protocol-specific differences are technical in origin, it is at least theoretically possible to train a generalizable model to remove these differences. Our results suggest this remains an opportunity for the development of novel correction methods suited for such a task.

## Data Availability

The original code used for this analysis, as well as the implementation of the existing R packages, is available on GitHub (https://github.com/Val554/10X_3prime_vs_5prime). The datasets analyzed for this study can be found in the CELLxGENE repository. Individual datasets are listed in Supplementary Table S1, along with CELLxGENE accession links. The three lists of biased genes can also be found in Supplementary Tables S8-S10.
