# Supplementary material for: Characterizing and mitigating protocol-dependent gene expression bias in 3′ and 5′ single-cell RNA sequencing
Source: Front Bioinform. 2026 Oct 8;6:1931243. doi: 10.3389/fbinf.2026.1931243 (PMC13687029; doi:10.3389/fbinf.2026.1931243)
Supplement: Supplementary file 1 [file Supplementaryfile1.docx]

Supplementary Material

# 1 Supplementary Figures and Tables

## 1.1 Supplementary Figures

**Supplementary Figure S1.** **Quality control and assay composition of the analyzed datasets.** Bar plots show the number of cells per subject, stratified by sequencing assay/batch, across the selected datasets: Suo (bone marrow, spleen, liver, kidney, and thymus), Park, Andrews, Leader, Simone, and Jardine. Within each dataset, panels correspond to individual subjects, and bars are colored by assay (red = 3′; blue = 5′). For five of the six datasets, the compared assays were 10X Genomics 3′ v2 and 10X Genomics 5′ v1. The Simone dataset instead compared 10X Genomics 3′ v3 and 10X Genomics 5′ v2. This distinction is indicated by the color of the box surrounding each dataset panel and the accompanying legend (blue outline: 3′ v2 vs 5′ v1; red outline: 3′ v3 vs 5′ v2).

**Supplementary Figure S2.** **Removal of the protocol-biased gene sets across datasets.** UMAP visualizations for the remaining datasets, Suo (bone marrow, spleen, liver, kidney, and thymus), Andrews, Simone, and Jardine, showing cells generated using the 10X 3′ (red) and 10X 5′ (blue) protocols. For each dataset, four UMAP plots display the original data as well as the same cells after removal of the 609, 867, and 2,598 biased gene sets (identified in Figure 1). For each plot, the value of the Seurat MixingMetric is indicated in gray below each plot, with higher values representing better batch mixing.

**Supplementary Figure S3.** **Removal of the protocol-biased gene sets across datasets.** UMAP visualizations for all datasets showing cells colored by cell type. For each dataset, two UMAP plots display the original data as well as the same cells after removal of the 867 biased genes (identified in Figure 1).

**Supplementary Figure S4. Cosine similarity of biased gene sets between 10X 3′ and 10X 5′ protocols across 36 matched subject samples.** Boxplots of cosine similarity between average gene expression profiles of 3′ and 5′ data, comparing scores for the **(A)** 609-gene list (1e^-100^ threshold) and the **(B)** 2,598-gene list (1e^-5^ threshold) to three replicates of randomly selected gene sets of equal size and expression proportion across all datasets. Cell types are shown as different shapes. Horizontal lines at 0 and 0.5 are shown to aid comparison across datasets and gene sets.

**Supplementary Figure S5. Validation of the 867 protocol-biased genes in matched 3′/5′ datasets.** Validation of the predefined 867 protocol-biased genes using seven matched 3′/5′ subject-level comparisons that were not used for biased-gene discovery. **(A)** Bar plots show the number of cells per subject, stratified by sequencing assay/batch, across the selected datasets: independent kidney validation dataset (Acera-Mateos et al.) and unused kidney, skin, and liver validation subjects from Suo et al. **(B)** UMAP visualizations of validation subjects before and after removal of the 867 protocol-biased genes. Cells are colored by assay/batch, and the Seurat MixingMetric is shown in grey below each plot, with higher values indicating better 3′/5′ mixing. **(C)** Cosine similarity between average 3′ and 5′ cell-type expression profiles calculated using the 867 biased genes and three expression-matched random gene sets. **(D)** Relationship between the number of cells and the number of significantly biased genes from the 867-gene list detected in each subject/sample at FDR < 0.05. Gray points indicate discovery/benchmarking subjects, and red triangles indicate validation subjects.

**Supplementary Figure S6. Identification and recurrence of protocol-biased genes across subjects. (A)** Number of genes significantly biased between 3′ and 5′ scRNA-seq libraries for each subject/dataset comparison at FDR <10^-50^. Subjects are shown on the y-axis and the number of significant genes is shown on the x-axis. Bar colors indicate the dataset of subject origin. The number in gray indicates the median number of 3′ and 5′ cells in the sample. **(B)** Number of 867 biased genes significant per subject at FDR < 10^-50^. **(C)** Recurrence of biased genes across subjects. Genes are ranked by the number of subjects in which they were significant. **(D)** Directionality of the 867 biased genes for each subject. Bars show the number of genes with higher expression in 3′ libraries versus higher expression in 5′ libraries. All genes in the list are shown without imposing a significance threshold. **(E)** Number of genes significantly biased between 3′ and 5′ scRNA-seq libraries in the F45_CD45P spleen subject (Suo et al., 2022) after quantification using exonic reads only or both exonic reads and intronic at FDR <10^-50^. **(F)** Number of 867 biased genes significant in the F45_CD45P spleen subject (Suo et al., 2022) after quantification using exonic reads only or both exonic and intronic reads at FDR <10^-50^. **(G)** Directionality of the 867 biased genes in the F45_CD45P spleen subject (Suo et al., 2022) after exonic-only or exonic-and-intronic quantification, shown for **(i)** all biased genes present and **(ii)** significant biased genes only at FDR < 10^-50^.

**Supplementary Figure S7. Characterization of detected genes and the 867 protocol-biased genes across the benchmark datasets. (A)** Proportion of genes detected in at least one of the six datasets **(i)** and distribution of undetected genes by gene biotype **(ii)**. Bar plot shows manually grouped Gene Ontology Biological Process terms enriched among undetected protein-coding genes **(iii)**. **(B)** Heatmap showing the number of 867 biased genes within each subject-level expression category. Genes with zero aggregate expression were assigned to Expr. 0, while expressed genes were divided into Q1 (lowest 25%), Q2 (25–50%), Q3 (50–75%), and Q4 (highest 25%). Rows are annotated by dataset and proportion of immune cells, shown as a white bar. **(C)** Gene Ontology enrichment of the 867 biased genes using all detected genes as background. Dot size represents gene count, and color represents adjusted p-value. **(D)** Number of non-immune-specific genes among the 867 biased genes and all detected genes. Non-immune-specific genes were detected in ≥30% of cells in at least one non-immune cell type and in <10% of cells in all immune cell types. **(E)** GC content within the first and last 250, 500, and 1000 bp of the longest protein-coding transcript for the 867 biased genes and expression-matched random genes. **(F)** Longest protein-coding transcript length distributions for 867 biased genes and three expression-matched random gene sets.

**Supplementary Figure S8. Extended benchmarking results of eleven common scRNA-seq batch correction methods for mitigating 10X 3′/5′ protocol bias.** Matched samples from 36 subjects across all datasets were independently corrected using each technique. **(A)** Boxplot of cosine similarity, MSE, and MSE without Z-transform, scVI, and scArches, comparing the normalization techniques across all datasets (shown as different shapes), with zero (red line) indicating the respective baseline. The dashed gray line indicates that the baseline used for ComBat through scArches is different from that of M3Drop and SCTransform (log-normalized vs log-normalized-scaled counts respectively). Higher cosine similarity and lower values for MSE denote better integration performance. Cosine similarity results are shown as relative percent change; MSE results are shown as percent change. Top shows the metrics for bias genes from Figure 1, bottom shows the metrics for the 3,000 Highly Variable Genes. **(B)** Boxplots showing the raw metric values underlying the relative comparisons in (A), including correlation coefficient, cosine similarity, Jensen–Shannon divergence, Euclidean distance, MSE, and MSE excluding selected methods (Z-transform, scVI, and scArches). For each method, values can be compared to their corresponding reference (“Log Normalize” or “Log Normalize Scaled”). The red outline highlights results computed using 867 biased genes, whereas the blue outline highlights results computed using the 3,000 HVGs.

**Supplementary Figure S9. Comparison of Scanorama-corrected expression values and Scanorama embeddings across representative datasets.** UMAP visualizations for six representative datasets showing 10X Genomics 3′ and 5′ data after Scanorama correction. The upper panel (in blue) shows UMAPs generated from the Scanorama-transformed gene expression, while the lower panel (in orange) shows UMAPs generated directly from Scanorama embeddings. Cells are colored by sequencing protocol (top) and by cell type (bottom – as identified by the authors). Only representative plots of datasets, with one subject per dataset, are shown.

**Supplementary Figure S10. Evaluation of eleven common scRNA-seq batch correction methods for preserving cell type structure after 10X 3′/5′ correction.** 36 matched samples from 6 datasets were independently integrated using each technique based on the workflow shown in Figure 2A. Boxplots show Seurat MixingMetric results by cell type for all datasets. Shapes represent different subjects. Higher values indicate greater mixing of cells across cell types, whereas lower values represent better preservation of cell-type separation.

**Supplementary Figure S11. Evaluation of eleven common scRNA-seq batch correction methods for preserving cell type markers.** Performance of eleven commonly used scRNA-seq batch correction methods was evaluated across multiple datasets for their ability to preserve cell type–specific gene expression signatures after correction of 10X Genomics 3′ and 5′ protocols. Each dataset is enclosed by a blue outline and displayed as a paired set of panels. For each dataset, the left panel shows boxplots of the Overlap Score across different cell types (indicated by distinct shapes). The Overlap Score quantifies the consistency of cell type marker identification between 10X 3′ and 10X 5′ data before and after batch correction, with higher values indicating greater agreement in marker recovery across protocols, as shown in Figure 2A. The right panel shows bar plots representing the number of overlapping cell type marker genes identified between 10X 3′ and 10X 5′ following correction. Log-normalized data serve as the reference baseline for comparison. Note that SCTransform and M3Drop automatically filter out some of the genes during QC.

**Supplementary Figure S12. Validation of ground truth DEGs across three use-case datasets and cell type comparisons.** Heatmaps display the expression patterns of ground truth DEGs identified between the two compared cell types in each dataset, created following the workflow described in Figure 4A. Results are shown for Park (thymus, n = 4 subjects), Suo (liver, n = 4 subjects), and Jardine (bone marrow, n = 3 subjects). For each dataset, rows correspond to the top 20 up- and down-regulated genes included in the ground-truth DEG set, and columns represent individual cells, grouped by cell type. Gene expression values are log-normalized within each dataset. The clear and consistent separation of expression patterns between the two cell types confirms that the selected ground truth genes represent cell type–specific markers and validates their use as a reference set for benchmarking integration performance.

## 1.2 Supplementary Tables

**Supplementary Table S1.** Description of datasets used in this study.

| **Dataset** | **Description** | **Tissue(s)** | **Technologies/ Batches**  **(Cell Ranger version)** | **Subjects Chosen** | **Total Cells** | **CELLxGENE accession link** |
| --- | --- | --- | --- | --- | --- | --- |
| Park (Park et al., 2020) | Human Thymic Development | Thymus | 10x 3′ v2, 10x 5′ v1  (v2.0.2 for 3′ and v2.1.0 for 5′) | “F29 45P”,  “F30 45P”, “F38 45P”,  “F41 45P”, “F41 45N”,  “F45 45P”, “F45 45N”,  “P1 CD3P”, “P1 CD3N” | 39,709 | <https://cellxgene.cziscience.com/collections/de13e3e2-23b6-40ed-a413-e9e12d7d3910> |
| Suo (Suo et al., 2022) | Human Immune System Across Organs | Bone marrow | 10x 3′ v2, 10x 5′ v1  (v3.0.2) | “F41_CD45P”, “F41_CD45N”, “F45_CD45P”,  “F45_CD45N” | 18,793 | <https://cellxgene.cziscience.com/collections/b1a879f6-5638-48d3-8f64-f6592c1b1561> |
|  |  | Kidney |  | “F41_CD45P” | 6,006 |  |
|  |  | Liver |  | “F32_CD45P”, “F34_CD45P”, “F38_CD45P”, “F41_CD45P”,  “F41_CD45N”, “F45_CD45N” | 25,983 |  |
|  |  | Spleen |  | “F45_CD45P”,  “F45_CD45N” | 17,187 |  |
|  |  | Thymus |  | “F38_CD45P”, “F41_CD45P”,  “F41_CD45N”, “F45_CD45P”,  “F45_CD45N” | 32,673 |  |
| Andrews (Andrews et al., 2024) | Human Liver | Liver | 10x 3′ v2, 10x 5′ v1  (v3.1.0) | “C59”, “C61”, “C63”, “C64” | 22,354 | <https://cellxgene.cziscience.com/collections/0c8a364b-97b5-4cc8-a593-23c38c6f0ac5> |
| Simone (De Simone et al., 2025) | Human Peripheral Blood Mononuclear Cells (PBMCs) | PBMCs | 10x 3′ v3, 10x 5′ v2  (v7.1.0)* | 4 samples (2 repeats per batch) from 1 subject | 29,944 | <https://cellxgene.cziscience.com/collections/398e34a9-8736-4b27-a9a7-31a47a67f446> |
| Leader (Salcher et al., 2022) | Human Non-Small Cell Lung Cancer | Lung (malignant and normal adjacent) | 10x 3′ v2, 10x 5′ v1  (v5.0.0) | 1 donor with two tissue samples: “Leader_Merad_2021_522_normal_adjacent”,  “Leader_Merad_2021_522_tumor_primary” | 11,755 | <https://cellxgene.cziscience.com/collections/edb893ee-4066-4128-9aec-5eb2b03f8287> |
| Jardine (Jardine et al., 2021) | Fetal Bone Marrow | Bone marrow | 10x 3′ v2, 10x 5′ v1  (v2.0.2 for 3′ and v2.11 for 5′) | “F30”, “F41”, “F45” | 11,569 | <https://cellxgene.cziscience.com/collections/793fdaec-5067-428a-a9db-ecefe135c945> |
| **Validation datasets** | | | | | | |
| Suo (Suo et al., 2022) | Human Immune System Across Organs | Kidney | 10x 3′ v2, 10x 5′ v1  (v3.0.2) | “F45_CD45N” | 3,390 | <https://cellxgene.cziscience.com/collections/b1a879f6-5638-48d3-8f64-f6592c1b1561> |
|  |  | Skin |  | “F45_CD45P”, “F38_CD45P” | 9,673 |  |
|  |  | Liver |  | “F45_CD45P” | 11,280 |  |
| Acera-Mateos (Acera-Mateos et al., 2026) | Cortex of kidney | Kidney | 10x 3′ v3  10x 5′ v1  (v5) | “20-687_N”  “20-688_N”  “PRECISE-38” | 9,664 | <https://cellxgene.cziscience.com/collections/4cbb929b-b03b-4aa8-a943-00f61dc22641> |

* Only Cell Ranger version 7 and above count intronic reads by default.

**Supplementary Table S2.** The original cell-type annotations for Andrews dataset and their respective coarse cell types used for further analysis. If the cell-type name is not in the table, its original representation was preserved.

| **Original Cell-Type Annotations** | **New Cell-Type Annotations** |
| --- | --- |
| CD4-positive, alpha-beta T cell,  CD8-positive, alpha-beta T cell,  natural killer cell | T/NK cell |
| centrilobular region hepatocyte,  midzonal region hepatocyte,  periportal region hepatocyte,  hepatocyte | hepatocyte |
| classical monocyte,  Kupffer cell,  monocyte,  macrophage | monocyte/macrophage |
| endothelial cell of artery,  endothelial cell of hepatic sinusoid,  endothelial cell of pericentral hepatic sinusoid,  endothelial cell of periportal hepatic sinusoid,  vein endothelial cell | endothelial |

**Supplementary Table S3.** The original cell-type annotations for Suo dataset (except Suo thymus) and their respective coarse cell types used for further analysis. If the cell-type name is not in the table, its original representation was preserved.

| **Original Cell-Type Annotations** | **New Cell-Type Annotations** |
| --- | --- |
| B-1 B cell,  B-2 B cell,  mature B cell,  immature B cell | B cell |
| CD8-alpha-alpha-positive,  alpha-beta intraepithelial T cell,  double-positive,  naive thymus-derived CD4-positive,  alpha-beta T cell,  naive thymus-derived CD8-positive,  regulatory T cell,  T cell  double negative thymocyte,  alpha-beta thymocyte | T cell |
| common dendritic progenitor,  dendritic cell,  plasmacytoid dendritic cell,  pre-conventional dendritic cell | dendritic cell |
| common myeloid progenitor,  early lymphoid progenitor,  granulocyte monocyte progenitor cell,  hematopoietic multipotent progenitor cell,  hematopoietic stem cell,  megakaryocyte-erythroid progenitor cell | hematopoietic progenitor cell |
| fraction A pre-pro B cell,  large pre-B-II cell,  late pro-B cell,  pro-B cell,  small pre-B-II cell | B cell progenitors |
| granulocyte,  myelocyte,  neutrophil,  promyelocyte | granulocyte |
| group 3 innate lymphoid cell,  group 2 innate lymphoid cell,  innate lymphoid cell | innate lymphoid cell |
| Kupffer cell,  Macrophage,  monocyte,  promonocyte | monocyte/macrophage |
| smooth muscle cell,  vascular associated smooth muscle cell | smooth muscle cell |

**Supplementary Table S4.** The original cell-type annotations for Leader dataset and their respective coarse cell types used for further analysis. If the cell-type name is not in the table, its original representation was preserved.

| **Original Cell-Type Annotations** | **New Cell-Type Annotations** |
| --- | --- |
| dendritic cell,  conventional dendritic cell,  CD1c-positive myeloid dendritic cell | dendritic cell |
| epithelial cell of lung,  club cell,  multiciliated epithelial cell,  pulmonary alveolar type 1 cell,  pulmonary alveolar type 2 cell | respiratory epithelial cell |
| bronchus fibroblast of lung,  fibroblast of lung | fibroblasts |
| smooth muscle cell,  pericyte | smooth muscle/pericyte cell |
| endothelial cell of lymphatic vessel,  capillary endothelial cell,  pulmonary artery endothelial cell,  vein endothelial cell | endothelial cell |

**Supplementary Table S5.** The original cell-type annotations for Jardine dataset and their respective coarse cell types used for further analysis. If the cell-type name is not in the table, its original representation was preserved.

| **Original Cell-Type Annotations** | **New Cell-Type Annotations** |
| --- | --- |
| precursor B cell,  immature B cell,  naive B cell | B cell |
| fraction A pre-pro B cell,  pro-B cell | B cell progenitors |
| CD4-positive, alpha-beta T cell,  CD8-positive, alpha-beta T cell,  regulatory T cell,  mature NK T cell,  CD16-negative, CD56-bright natural killer cell, human,  natural killer cell,  immature natural killer cell | T/NK cell |
| common dendritic progenitor,  dendritic cell | dendritic cell |
| myelocyte,  neutrophil,  promyelocyte,  eosinophil,  basophil | granulocyte |
| endothelial cell of sinusoid,  endothelial tip cell,  endothelial cell | endothelial cell |
| muscle cell,  muscle precursor cell,  precursor cell,  chondrocyte,  fibroblast,  myofibroblast cell | stromal/mesenchymal lineage |
| primitive red blood cell,  erythrocyte | RBCs |

**Supplementary Table S6.** Description of batch correction methods used in this study.

| **Normalization (*function name*)** | **Input** | **Output** | **Memory** | **Runtime** | **Reference package version** |
| --- | --- | --- | --- | --- | --- |
| ComBat (*ComBat*) | Expression matrix of log-normalized counts from the “data” layer of the Seurat object.  Parameters:  > **batch** set to the metadata column indicating batch (“assay”) | Corrected expression matrix. | 16.10 GB | 1 min 51 sec | Johnson et al. (Johnson et al., 2007), sva package version 3.54.0 |
| Limma (*removeBatchEffect*) | Expression matrix of log-normalized counts from the “data” layer of the Seurat object.  Parameters:  **> batch** set to the metadata column indicating batch (“assay”) | Corrected expression matrix. | 16.23 GB | 57 sec | Ritchie et al. (Ritchie et al., 2015), limma package version 3.62.2 |
| mnnCorrect (*mnnCorrect*) | Expression matrix of log-normalized counts from the “data” layer of the Seurat object.  Parameters:  > **batch** set to the metadata column indicating batch (“assay”)  > **subset.row** set to the list of HVGs | SingleCellExperiment object containing the “corrected” counts. The counts can be extracted from the object and loaded into an original Seurat object, if desired. | 30.55 GB when run in parallel for each sample | 51 min 23 sec  When run in parallel for each sample | Haghverdi et al. (Haghverdi et al., 2018), batchelor package version 1.22.0 |
| fastMNN (*fastMNN*) | Expression matrix of log-normalized counts from the “data” layer of the Seurat object.  Parameters:  > **batch** set to the metadata column indicating batch (“assay”)  > **subset.row** set to the HVGs  > **correct.all** = TRUE | Corrected expression matrix. | 15.76 GB | 2 min 4 sec | Haghverdi et al. (Haghverdi et al., 2018), batchelor package version 1.22.0 |
| Scanorama* (*scanorama.correct_scanpy*) | List of scanpy.AnnData objects split by the batch (“assay”) with log-normalized counts in their adata.X:  > **return_dimred** = TRUE  > **approx** = FALSE | AnnData objects where adata.X is replaced with the Scanorama-transformed expression matrix and obsm['X_scanorama'] containing the low-dimensional embeddings. | 5.1 GB | 34 sec | Hie et al. (Hie et al., 2024),  Scanorama package version 1.7.4 |
| Z-transform | Custom-made function that z-transforms the expression matrix of log-normalized counts from the “data” layer of the Seurat object. Done separately for each batch and merged at the end, if desired. | Corrected expression matrix. | 17.98 GB | 49 sec | N/A |
| scVI* (*scvi.model.SCVI*) | AnnData object with **raw** counts in adata.X:  For *setup_anndata*: > **layer** is “counts” > **batch_key** is set to the metadata column indicating batch (“assay”)  Then the model is set and trained with desired hyperparameters and early stopping. | The corrected expression can be extracted from the trained model using *model.get_normalized_expression*: > **transform_batch** is set to one of the desired batches to cast all the data to > **library_size** is set to reflect the data | 3.85 GB | 45 min 49 sec | Lopez et al. (Lopez et al., 2018), scVI package version 1.3.1 |
| scArches* (*scarches.model.SCVI*) | AnnData object with **raw** counts in adata.X:  1) *prepare_query_anndata*  2) *load_query_data*:  > **freeze_dropout** = TRUE  3) then *train*:  > **plan_kwargs** = dict(weight_decay=0.0) | The corrected expression can be extracted from the trained model using *model.get_normalized_expression*: > **transform_batch** is set to one of the desired batches to cast all the data to > **library_size** is set to reflect the data | 4.94 GB | 54 min 51 sec | Lotfollahi et al. (Lotfollahi et al., 2022), scarches package version 0.6.1 |
| M3Drop (*NBumiConvertData*,  *NBumiPearsonResiduals*) | Expression matrix of **raw** counts extracted from the “counts” layer of the Seurat object. It does not have a parameter for the batch, so expression matrix for each batch must be passed separately and merged at the end, if desired. | Corrected expression matrix. **Not all genes are returned.** | 16.31 GB | 1 min 25 sec | Andrews and Hemberg (Andrews and Hemberg, 2019), M3Drop package version 1.32.0 |
| SCTransform (*SCTransform*) | Seurat object with **raw** counts in the “counts” layer.  Parameters:  > **vars.to.regress** set to the metadata column indicating batch (“assay”)  > **residual.features** set to the HVGs  > **return.only.var.genes** = FALSE | Seurat object with normalized counts in “SCT” assay, layer “scale.data”. **Not all genes are returned.** | 7.04 GB if *glmGamPoi* is additionally installed from BiocManager (58) | 1 min 29 sec if *glmGamPoi* is additionally installed from BiocManager (58) | Hafemeister and Satija (Hafemeister and Satija, 2019), Seurat package version 5.2.1 |
|  | Seurat object with **raw** counts in the “counts” layer is split by the batch column (using Seurat v5 *split* function).  > **residual.features** set to the HVGs  > **return.only.var.genes** = FALSE |  | 10.07 GB if *glmGamPoi* is additionally installed from BiocManager (58) | 2 min  3 sec if *glmGamPoi* is additionally installed from BiocManager (58) |  |

* Methods run on an Apple M3 Pro chip

**Supplementary Table S7.** Description of cell-type sparsification strategy used for the biological use-case. Each column indicates which cell types were retained for each protocol.

| **Dataset** | **Only 3**′ | **Only 5**′ | **Both 3**′ **and 5**′ |
| --- | --- | --- | --- |
| Park (thymus) | CD8-positive, alpha-beta T cell,  gamma-delta T cell,  T-helper 17 cell,  double negative thymocyte,  thymocyte | regulatory T cell,  T cell,  double-positive, alpha-beta thymocyte | CD4-positive, alpha-beta T cell,  alpha-beta T cell |
| Suo (liver) | dendritic cell,  endothelial cell,  granulocyte | natural killer cell | B cell progenitors,  B cell,  hematopoietic progenitor cell,  innate lymphoid cell,  T cell |
| Jardine (bone marrow) | dendritic cell,  early lymphoid progenitor,  macrophage,  promonocyte,  RBCs | B cell,  mast cell,  megakaryocyte,  osteoclast | granulocyte monocyte progenitor cell,  B cell progenitors |
